# Deblurring Ghost Imaging Reconstruction Based on Underwater Dataset Generated by Few-Shot Learning

**DOI:** 10.3390/s22166161

**Published:** 2022-08-17

**Authors:** Xu Yang, Zhongyang Yu, Pengfei Jiang, Lu Xu, Jiemin Hu, Long Wu, Bo Zou, Yong Zhang, Jianlong Zhang

**Affiliations:** 1 School of Information Science and Technology, Zhejiang Sci-Tech University, Hangzhou 310018, China; 2 Institute of Land Aviation, Beijing 101121, China; 3 Institute of Optical Target Simulation and Test Technology, Harbin Institute of Technology, Harbin 150001, China

**Keywords:** few-shot learning, underwater deblurring ghost imaging, paired underwater datasets, low sampling rate

## Abstract

Underwater ghost imaging based on deep learning can effectively reduce the influence of forward scattering and back scattering of water. With the help of data-driven methods, high-quality results can be reconstructed. However, the training of the underwater ghost imaging requires enormous paired underwater datasets, which are difficult to obtain directly. Although the Cycle-GAN method solves the problem to some extent, the blurring degree of the fuzzy class of the paired underwater datasets generated by Cycle-GAN is relatively unitary. To solve this problem, a few-shot underwater image generative network method is proposed. Utilizing the proposed few-shot learning image generative method, the generated paired underwater datasets are better than those obtained by the Cycle-GAN method, especially under the condition of few real underwater datasets. In addition, to reconstruct high-quality results, an underwater deblurring ghost imaging method is proposed. The reconstruction method consists of two parts: reconstruction and deblurring. The experimental and simulation results show that the proposed reconstruction method has better performance in deblurring at a low sampling rate, compared with existing underwater ghost imaging methods based on deep learning. The proposed reconstruction method can effectively increase the clarity degree of the underwater reconstruction target at a low sampling rate and promotes the further applications of underwater ghost imaging.

## 1. Introduction

The mainstream technology of underwater imaging is sonar imaging based on acoustic waves. However, due to the long wavelength of an acoustic wave, sonar imaging has difficulty overcoming the gap of the multipath effect and low imaging resolution, which limits the development of sonar imaging in the field of underwater target imaging. Due to high imaging resolution, underwater active optical imaging [1] has become an important technology for underwater imaging. With the development of underwater active optical imaging, more and more underwater active optical imaging approaches have been proposed to improve the quality of the underwater imaging and decrease the noise of reconstructed results at a low sampling rate. In order to reduce the backscattering, polarimetric imaging technology [2,3] was developed by Liu, F. et al. to solve the problem. However, it also blocks some of the light that hints the detector, which decreases the quality of the underwater imaging results. Range-gated imaging technology [4] was developed by Mariani et al. to eliminate the backscattering effect. However, due to the high complexity of underwater environments, the reconstructed results based on conventional underwater imaging technology are facing low contrast, fuzzy details, information loss, color distortion, and many other problems. It has become a research focus of underwater vision and underwater image processing to improve the quality of underwater imaging.

Recently, ghost imaging (GI) [5] has attracted much more attention. Compared to the traditional underwater imaging technology including polarimetric imaging and range-gated imaging, GI technology has the advantages of long imaging distance, high resolution, high detection efficiency, low noise, strong anti-interference ability, easy integration, and miniaturization. GI was first proposed by Pittman et al. in 1995 with entangled photon pairs [6]. Since then, GI has been a popular topic in many fields, such as remote sensing [7], fluorescence imaging [8], terahertz imaging [9], lidar [10], etc. In 2008, Shapiro proposed the computational GI scheme with a single path [11], where the reference path is removed. Since then, more and more GI improvement schemes have been applied, such as iterative denoising GI [12], scalar matrix structure with GI [13], differential GI [14], and Hadamard GI [15].

However, enormous measurements are needed to reconstruct an image with high quality, which will increase the time of sampling and decrease the efficiency of the experiment. In order to solve the issue, more and more highly effective reconstruction methods are being developed. Compressive Sensing Ghost Imaging (CSGI) [16,17] has been demonstrated to be a useful method to solve the issue. CSGI can reconstruct images with high quality and intensity at a low sampling rate by utilizing the sparse characteristics of the image in the orthogonal variable domain. Owing to these improvements, CSGI has been used to reconstruct underwater objects. However, CSGI demands a long time to reconstruct an underwater image [18]. In addition, the underwater image reconstruction by compressed sensing needs a lot of computing resources, which also leads to poor real-time data processing in practical applications. Although CSGI can achieve the purpose of a low sampling rate to a certain extent, enormous measurements are still required to reconstruct high-quality images. Therefore, the contradiction between the number of measurements and image quality is still very large.

Deep learning, which has become popular in machine vision, has been used in the field of image reconstruction. In recent years, more and more methods have applied deep learning in GI [19,20,21,22,23,24,25] to reconstruct high-quality results at low sampling rates. In early studies, the input image of the GI based on deep learning was obtained by conventional GI. In 2019, Wang et al. proposed a neural network for CGI [22] which reconstructs the target image directly from the one-dimensional signal collected by the bucket detector [26]. Compared to previous methods, deep learning algorithms are more efficient and require fewer measurements for reconstruction. Based on these, more and more people applied deep learning in underwater ghost imaging [27].

However, in order to reconstruct high-quality results, the training of the underwater ghost imaging requires enormous paired underwater datasets, but it is difficult to obtain paired underwater datasets directly. Although the Cycle-GAN method can solve the problem of dataset mismatch to some extent, there exist two problems. (1) The training of Cycle-GAN requires a huge amount of underwater blurring data. (2) The blurring degree of the fuzzy class of the paired underwater datasets generated by the Cycle-GAN is unitary. Due to the disadvantages of Cycle-GAN, it cannot meet the training requirements of the reconstructed network. In order to solve these problems, the few-shot underwater image generative network (FUIGN) method based on the FUNIT method is proposed in this study. The FUIGN method can generate a paired “Clear-Fuzzy” dataset under the condition that the number of real underwater fuzzy datasets is relatively small. Specifically, the proposed FUIGN is trained with the asymmetric underwater dataset, which contains 15,000 underwater clear images and 1500 underwater fuzzy images. In addition, one part of the training datasets was collected by a web crawler and the other part was collected from ImageNet [28]. The number of fuzzy images is rather smaller than that of clear images, which is a common problem of few-shot image to image translation. Meanwhile, in the generated paired dataset, a clear image is corresponding to a large number of fuzzy images with different blurring degrees. Therefore, the fuzzy degree diversity of the generated images is high, which is convenient for the reconstructed network to learn how to remove various fuzzy features. It is helpful to utilize the paired underwater datasets generated by the proposed method to train the network model to improve the generalization ability of the reconstructed network model. The simulation results show that the paired underwater datasets generated by FUIGN are better than those by the Cycle-GAN method, which meets the training requirements of the reconstructed network.

To reconstruct results with a high clarity degree, an underwater deblurring ghost imaging based on few-shot (UDGI-FS) at a low sampling rate is proposed to increase the clarity degree of the underwater imaging. The UDGI-FS consists of two parts: reconstruction and deblurring. The reconstruction adopts the underwater ghost imaging based on deep learning (UGI-DL) to reconstruct the underwater image, and the deblurring adopts the $G_{B2A}^{\left(F\right)}$ generator of FUIGN to decrease the blurring degree of the reconstructed results of the UGI-DL method. The generator of UGI-DL adopts a modified U-Net with res-net block and double skip connections. The attention gate is added in each skip connection. The input of the generator of UGI-DL is a 1D signal recorded by the bucket detector and the output of the generator is the reconstructed underwater image. Then, the $G_{B2A}^{\left(F\right)}$ of FUIGN is used to further decrease the blurring degree of the output of the generator of UGI-DL. Simulation and experimental results demonstrate that the reconstructed underwater results by the UDGI-FS method trained with the paired underwater datasets have a high clarity degree and high generation ability. The proposed UGI-FS is suitable for the reconstruction of an underwater image.

In general, the contribution of this work is mainly in three aspects:

(1) The FUIGN method is proposed to obtain paired underwater datasets whose fuzzy class contains underwater images with different blurring degrees. Training FUIGN requires only the asymmetric underwater dataset, which reduces the amount of real underwater fuzzy data.
(2) The UDGI-FS method is proposed to obtain reconstructed underwater results with high quality. The reconstruction method consists of two parts: UGI-DL used to reconstruct the underwater image and the $G_{B2A}^{\left(F\right)}$ used to decrease the blurring degree of the output of UGI-DL. The generator of UGI-DL adopts a modified U-Net with res-net block and double skip connections, and the attention gate is added in each skip connection. The $G_{B2A}^{\left(F\right)}$ is the generator of FUIGN.
(3) Simulation and experimental results demonstrate that the paired underwater datasets generated by FUIGN are better than those of the Cycle-GAN method, which meets the training requirements of the UDGI-FS. In addition, the reconstructed underwater results generated by the UDGI-FS method at a low sampling rate have a high clarity degree, which indicates that the proposed UGI-FS is suitable for the reconstruction of an underwater image.

## 2. Method

### 2.1. UDGI-FS Imaging Scheme

**Figure 1:**
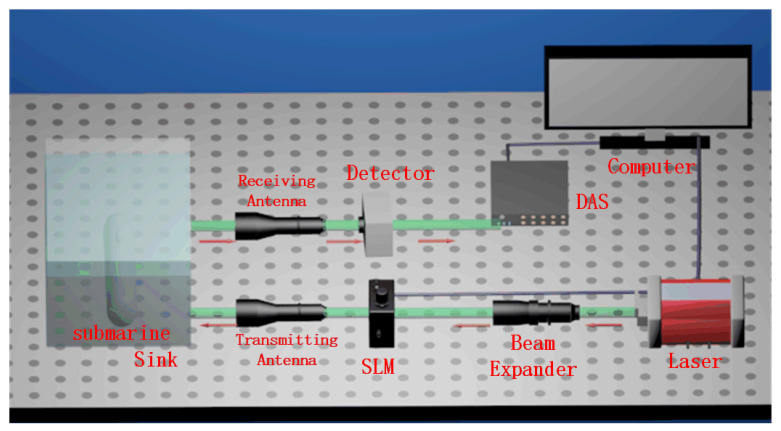
Schematic of UDGI-FS system.

Figure 1 shows the UDGI-FS system scheme. The light source emits the light to the beam expander which can expand the light. Then, the spatial distribution of the light is modulated by the spatial light modulator (SLM) [29] according to the pre-programmed random speckle modulation modes, which are in the form of a 128 × 128 matrix. The modulated laser emits on the target in the sink. The reflected laser is collected by a bucket detector to measure the transmission intensity of each pattern. Then, the corresponding light intensity is collected by the data acquisition system (DAS) and sent to the computer via USB for reconstruction. 

### 2.2. Theory and Forward Imaging Model

In the paper, the simulated underwater object is a two-dimensional image. The reflectivity distribution of the object can be expressed as *T* (*x*, *y*), where (*x*, *y*) are the pixel coordinates. The random speckle which is illuminated on the object can be denoted by *I_m_*. The reflected light is focused on the bucket detector. Hence, the *m*th measurement of bucket detector can be written as:

(1) $$S_{m}=\sum_{\left(x,y\right)}T\left(x,y\right)I_{m}\left(x,y\right).$$

According to the GI theory, the underwater object image *T* (*x*, *y*) can be restored by a second-order intensity fluctuation correlation of the total light intensity with random speckle patterns. Therefore, the reconstructed object image *T*′ (*x*, *y*) can be written as:

(2) $$T^{\prime }(x,y)=<S_{m}I_{m}>-<S_{m}><I_{m}>.$$

where < > denotes an assemble average.

The proposed method UDGI-FS trained by the paired underwater datasets is used to reconstruct the underwater object image. Its reconstruction process can be expressed as:

(3) $$x^{*}=DL\left\{y\right\}.$$

where *DL*{.} represents the trained neural network. The **y** and x* are the input and output of the *DL*{.}, respectively. The paired underwater datasets are used to train the neural network *DL*{.}. The network structure can be written as:

(4) $$DL_{learn}=\underset{DL_{\theta },\theta \in \Theta }{\arg\min}\sum_{j=1}^{J}L\left(x^{\left(j\right)},DL_{\theta }\left\{y^{\left(j\right)}\right\}\right)+\varphi \left(\theta \right).$$

where the **x**^(*j*)^ and **y**^(*j*)^ are the target image and the *M* one-dimensional measurements, respectively, *L*(.) is the loss function, *J* is the total number of the dataset, and Θ is the set of all possible parameters of the neural network. The function of the *φ*(*θ*) is used to avoid overfitting [30].

### 2.3. Preparation of Paired Training Dataset with FUIGN

Different from the conventional ghost imaging based on deep learning trained with the dataset captured in free space, the proposed UDGI-FS neural network needs the paired underwater datasets to train. The paired underwater datasets include two classes of dataset: underwater clear dataset serving as the object image for network training and underwater fuzzy dataset serving as an experimental object. However, it is hard to directly obtain paired underwater datasets. In order to solve the problem, the Cycle-GAN neural network [31] is proposed to generate paired underwater datasets through the style transform approach. The experimental and simulation results show that the Cycle-GAN method can solve the above problems to some extent. However, the fuzzy underwater dataset generated by Cycle-GAN is facing the problem that the blurring degree of the underwater dataset is unitary, which will decrease the generation ability of the UDGI-FS. The reason is that the input of the generator of Cycle-GAN is only the source domain image, which cannot instruct the generator to produce an underwater image with different blurring degrees.

In order to obtain an underwater fuzzy dataset with different blurring degrees, the FUIGN method based on the FUNIT method [32] is proposed. The FUIGN method is used to conduct style migration and transformation, which can be used to generate paired underwater datasets. In this paper, the style of the underwater dataset is the blurring degree. The dataset for training the FUIGN method contains two classes of datasets, including an underwater clear dataset and underwater fuzzy dataset. The underwater clear dataset with 15,000 underwater images and the underwater fuzzy dataset with 1500 underwater images are crawled from the internet. The fuzzy dataset contains different blurring degrees of the underwater dataset.

The input of the generator of FUIGN contains two classes of images: content class with clear images and style class with fuzzy images. The style class is the key that can control the blurring degree of the output image. The goal of FUIGN is to map an underwater image of content class to an underwater image of style class in terms of the blurring degree of the style class.

Specifically, two generators, $G_{A2B}^{\left(F\right)}$ and $G_{B2A}^{\left(F\right)}$, are used to generate paired underwater datasets. The function of the generator $G_{A2B}^{\left(F\right)}$ is to convert the clear image *I_A_* to the fuzzy image *I_A_*_2*B*_ which should have the same blurring degree of the fuzzy image *I_B_*, which will solve the problem of unitary blurring degree. The function of the generator $G_{B2A}^{\left(F\right)}$ is to convert the fuzzy image *I_B_* to the clear image *I_B_*_2*A*_ which should have the same blurring degree with the clear image *I_A_*, which will further improve the quality of the reconstructed results of UGI-DL. The experimental and simulation results verify the effectiveness of the proposed FUIGN method.

**Figure 2:**
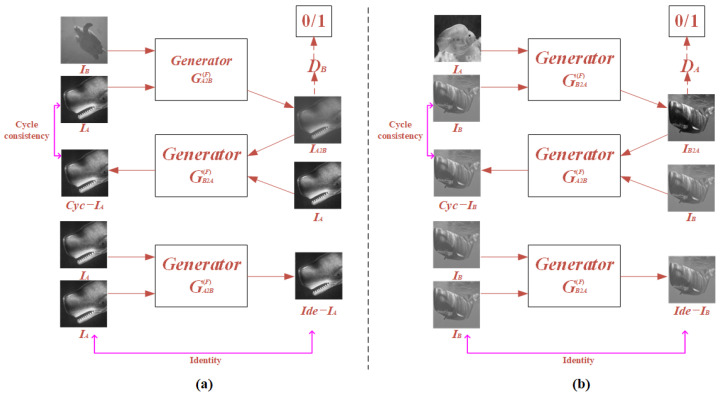
Schematic of FUIGN.

The scheme of the FUIGN method is shown in Figure 2. As Figure 2 shows, FUIGN consists of two generators and two discriminators. The training of FUIGN adopts the method of unsupervised learning, which will have a broad application prospect. Figure 2a shows the generative adversarial model ($G_{A2B}^{\left(F\right)}$) converting from class A to class B. As Figure 2a shows, the content image *I_A_* and style image *I_B_* are the inputs of the generator $G_{A2B}^{\left(F\right)}$. *I_A_*_2*B*_, which should have the same blurring degree as the style image *I_B_*, is the output of the $G_{A2B}^{\left(F\right)}$. Then, *I_A2B_* is the input of the generator $G_{B2A}^{\left(F\right)}$ and the output of $G_{B2A}^{\left(F\right)}$ is *Cyc*-*I_A_*, which should have the same blurring degree as the content image *I_A_*. The process is called cycle consistency, used to ensure the integrity of the content information of the output image. Meanwhile, *I_A_*_2*B*_ is judged by the discriminator D_B_ to be determined a real image or a generated image. The *I_de_*−*I_A_* is the generated image by the $G_{A2B}^{\left(F\right)}$. The process is called identity and is used to ensure that the texture information of the content image and output should be consistent. 

Similarly, Figure 2b shows the generative adversarial model $G_{B2A}^{\left(F\right)}$ converting from class B to class A. The process of Figure 2b is similar to Figure 2a.

**Figure 3:**
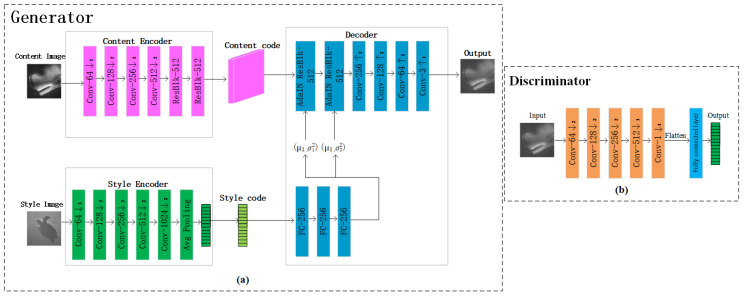
Generator and discriminator of FUIGN. (**a**) architecture of the generator of FUIGN; (**b**) architecture of the discriminator of FUIGN.

The proposed FUIGN network structure is shown in Figure 3. The network structure of FUIGN is presented below.

Generator: As the top network structure chart of Figure 3 shows, the generator takes one content image and one style image as inputs. The generator is composed of two encoders, a content encoder and style encoder, and one decoder. The content encoder extracting structural information of the content image is composed of four 2D convolution layers followed by two residual blocks. The style encoder extracting style information of the style image is composed of five 2D convolution layers followed by one average pooling layer. The decoder takes two inputs, the content code and style code. The content code goes through two adaptive instance normalization residual blocks followed by four upscale convolution layers. The style code is merged to the content code, by taking the affine transformation of the features from a series of fully connected layers. The affine transformation acts globally on the content image, thereby preserving its structure. After training, the generator only needs two images, the content image and one is the style image, to generate the output image during the test time.

Discriminator: As the bottom structure chart of Figure 3 shows, the discriminator is composed of five 2D convolution layers and a fully connected layer. The five convolution layers are used to extract the features of the input underwater image. The last fully connected layer is used to transform the output of the five convolution layers into a one-dimensional eigenvector to output, which will easily achieve the goal of discrimination.

The loss function is used to improve the reconstruction quality of the generator. The total loss of the generator can be expressed as:

(5) $$L_{\mathrm{total}}=\alpha L_{\mathrm{adv}}+\beta L_{\mathrm{cyc}\;}+\gamma L_{\mathrm{ide}}.$$

where the *L*_adv_, *L*_cyc__,_ *L*_ide_ refer to the adversarial loss, cycle consistency loss, and identity loss. The *α*, *β*, *γ*, the weighting coefficients of *L*_adv_, *L*_cyc__,_ *L*_ide_, respectively, are 1, 10, and 10, respectively.

**Adversarial loss:** The adversarial loss has the functions for the discriminator to distinguish real images from the output images of generator and for the generator to fool the discriminator by generating images as real images. Adversarial loss can be described as:

(6) $$L_{\mathrm{adv}}=\frac{1}{B}\sum_{b=1}^{B}\left({\left(D_{B}(G_{A2B}(I_{A},I_{B}))-1\right)}^{2}+{\left(D_{A}(G_{B2A}(I_{B},I_{A}))-1\right)}^{2}\right).$$

where *B* is batch size.

**Cycle consistency loss:** The cycle consistency loss [31] is used to ensure the integrity of structural information of the content image, which will ensure that the output image of generator has the same structure as the content image. The cycle consistency loss can be described as:

(7) $$L_{\mathrm{cyc}}=||G_{B2A}(G_{A2B}(I_{A},I_{B}),I_{A})-I_{A}|{|}_{1}+||G_{A2B}(G_{B2A}(I_{B},I_{A}),I_{B})-I_{B}|{|}_{1}.$$

**Identity loss:** The identity loss is used to ensure that the texture information of the input content image and output image of the generator is consistent. The identity loss can be described as:

(8) $$L_{\mathrm{ide}}=||G_{A2B}(I_{A},I_{A})-I_{A}|{|}_{1}+||G_{B2A}(I_{B},I_{B})-I_{B}|{|}_{1}.$$

### 2.4. Reconstruction of Network Structure

**Figure 4:**
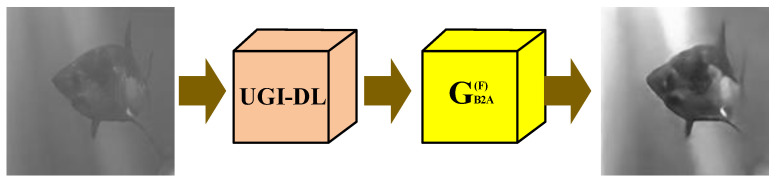
UDGI-FS consists of UGI-DL and $G_{B2A}^{\left(F\right)}$.

**Figure 5:**
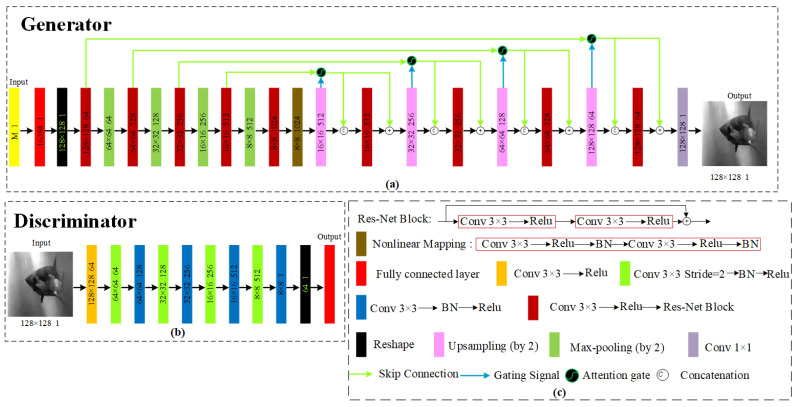
UGI-DL consists of a generator G and a discriminator D. (**a**) architecture of the generator of UGI-DL; (**b**) architecture of the discriminator of UGI-DL; (**c**) annotations of generator and discriminator of UGI-DL.

The network structure of the proposed UDGI-FS is shown in Figure 4. The UDGI-FS consists of two parts: UGI-DL and $G_{B2A}^{\left(F\right)}$_._ First, UGI-DL is used to reconstruct the underwater image. Then, the generator $G_{B2A}^{\left(F\right)}$ of FUIGN is used to decrease the blurring degree of the output of UGI-DL. The network structure of $G_{B2A}^{\left(F\right)}$ is the top network structure chart of Figure 3. The network structure of UGI-DL is shown in Figure 5.

The proposed FUIGN network structure is shown in Figure 5.

**Generator:** Inspired by UGI-GAN [27], attention U-Net [33,34,35], and Res-Net [36], the modified generator is proposed. As the top network structure of the chart of Figure 5 shows, the generator takes a one-dimensional vector with a size of *M* × 1 as input. As the highest sampling rate in the experiment is 20%, the value of *M* is less than 3277. The following two layers, the fully connected layer and reshape layer, are used to reshape the one-dimensional vector to a channel underwater image size of 128 × 128. A pair of digits in the format of ‘128 × 128 1’ is placed at each module, where 128 × 128 denotes the size of the underwater image and 1 denotes the number of the channel of the underwater image. The following network is a modified U-Net structure consisting of an encoder and a decoder. The main function of the encoder is to extract the feature image, while the main function of the decoder is to recover the feature image. The encoder uses the convolutional layers to extract the feature information, structural information, and content information of the underwater image. Each convolutional layer is followed by a res-net block, as shown in Figure 5c. Every res-net block is composed of two 2D convolution layers. The res-net block is used to accelerate the training of the network model and improve the generation ability of the neural network. In addition, every convolutional layer is followed by a max-pooling layer. Furthermore, the double connection is used to connect the encoder-decoder to achieve a better reconstruction effect. In addition, to each skip connection is added an AG [37] to filter noise, and in order to further improve the ability of generalization, the nonlinear mapping layer is added between the encoder and decoder, as shown in Figure 5c. In a word, the generator of UGI-DL uses double skip connections with adding AG in each skip connection and a nonlinear mapping layer to reconstruct object image with high quality, which is better than UGI-GAN.

**Discriminator:** As the bottom structure chart of Figure 5 shows, the discriminator is composed of nine 2D convolution layers and a fully connected layer. The nine convolution layers are used to extract the features of the input underwater image. The last fully connected layer is used to transform the output of the nine convolution layers into a one-dimensional eigenvector to output, which will easily achieve the goal of discrimination.

The training process of the network is to optimize the parameters in the set Θ including weights and bias in two neighboring layers. The Adam optimizer [38] is used as the main optimizer to optimize the network parameters. The epoch and batch size are set as 100 and 48, respectively. The initial learning rate (LR) is 0.0001, and when the epoch increases to 50, the LR decreases to 0.00001.

Learning rate (LR): The parameters of the model are relatively random at the beginning. A relatively large learning rate was chosen, resulting in a faster decrease in the loss. Therefore, LR was set to 0.0001. After a period of training, the value of the loss function hovers around the minimum value and fluctuates greatly. It is always difficult to reach the optimal value. Therefore, the method of learning rate decay [39] should be used and the learning rate dropped from 0.0001 to 0.00001 after 50 epochs, which could avoid overfitting. Batch size: A single 2080-Ti GPU used to accelerate the computation can support 48, the maximum value of the batch size. Therefore, the batch size was set to 48. Epoch: In the process of network training, the loss decreases with the increase in the number of the epoch. By observation, the loss tends to stabilize as the number of the epoch approaches 100. Therefore, the epoch was set to 100.

The loss function is used to improve the reconstruction quality of the generator. The total loss of the generator can be expressed as:

(9) $$L_{\mathrm{total}\;}=\alpha L_{\mathrm{perceptual}\;}+\beta L_{\mathrm{pixel}\;}+\gamma L_{\mathrm{adversarial}\;}.$$

where the *L*_perceptual_, *L*_pixel__,_ *L*_adversarial_ refer to the perceptual loss, pixel loss, and adversarial loss. The *α*, *β*, *γ*, the weighting coefficients of *L*_perceptual_, *L*_pixel__,_ *L*_adversarial_ respectively, are 0.006, 1, and 0.001, respectively.

The function of the *L*_pixel_, which plays the main role in the training of the network model, is to enable the network model to generate high-quality reconstructed images. The function of the other two loss functions, which play the supporting roles in the training of the network model, is to further help the network model generate high-quality reconstructed images. Therefore, *β*, the weighting coefficient of *L*_pixel_, is set to 1, while *α* and *γ*, the weighting coefficients of *L*_perceptual_ and *L*_adversarial_, respectively, are set to 0.006 and 0.001, respectively.

In the training process, the mean square error (MSE) is implemented in loss functions. Perceptual loss and pixel loss can be described as:

(10) $$L_{\mathrm{perceptual}\;}=\frac{1}{BHV}\sum_{b=1}^{B}\sum_{h=1}^{H}\sum_{v=1}^{V}{\left(f_{\mathrm{vgg}19}(x_{g})-f_{\mathrm{vgg}19\;}(x)\right)}^{2}.$$

(11) $$L_{\mathrm{pixel}\;}=\frac{1}{BHV}\sum_{b=1}^{B}\sum_{h=1}^{H}\sum_{v=1}^{V}{\left(x_{g}-x\right)}^{2}.$$

where *x_g_* and *x* are the reconstructed image and the real underwater image, respectively, and H, V are the width and height of *x_g_* and *x*. *B* is the batch size. In addition, *f*_vgg19_ is the trained VGG19 network [40].

Adversarial loss can be described as:

(12) $$L_{adversarial}=\frac{1}{B}\sum_{b=1}^{B}{\left(D(G(y))-1\right)}^{2}.$$

where *y* is the input of the network model. *G*(*y*) is the output of the generator. 

The model was trained on a python 3.8 version with PyTorch 1.5.1. A single 2080-Ti GPU was used to accelerate the computation.

Under the same condition, the durations for the training and reconstruction of the three methods at 20% sampling rate are shown in Table 1. As Table 1 shows, the training times of the three methods are about the same. The reconstruction duration was the average time of all test images. As Table 1 shows, although the reconstruction duration of UDGI-FS is higher than that of the other two methods, the imaging quality of the UDGI-FS method is better than that of the other two methods.

## 3. Numerical Simulation Results

### 3.1. Generate Dataset Comparisons

**Figure 6:**
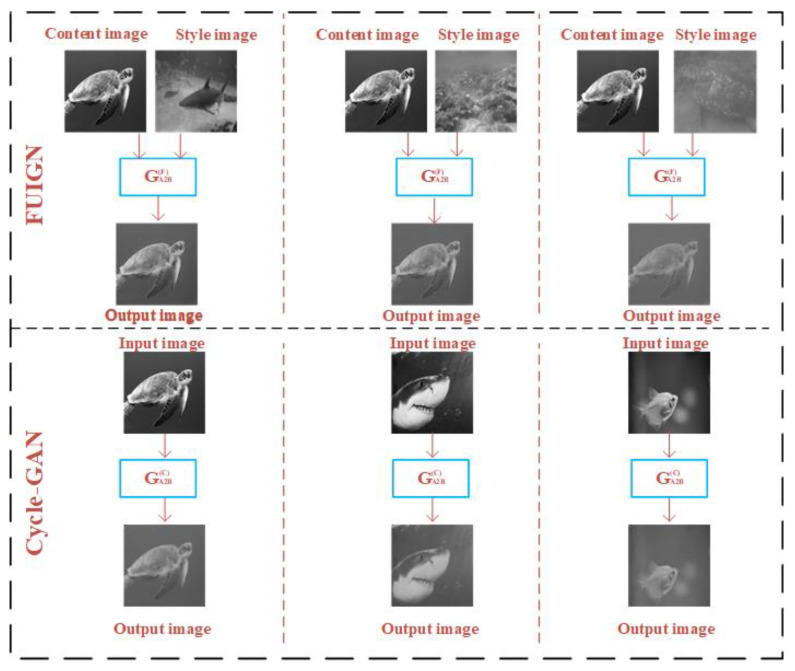
Comparison of the underwater paired images generated by the $G_{A2B}^{\left(F\right)}$ of FUIGN and $G_{A2B}^{\left(C\right)}$ of Cycle-GAN.

**Figure 7:**
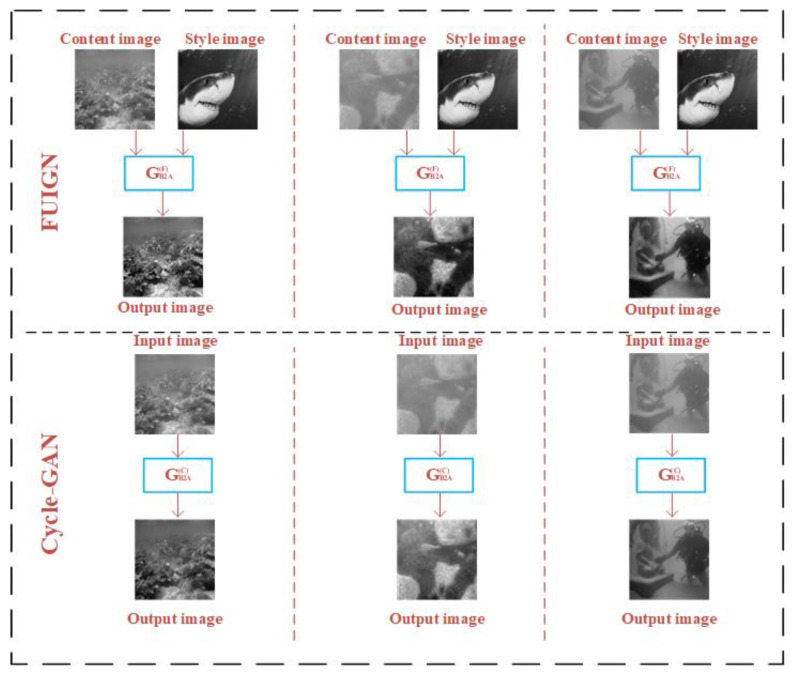
Comparison of the underwater paired images generated by the $G_{B2A}^{\left(F\right)}$ of FUIGN and $G_{B2A}^{\left(C\right)}$ of Cycle-GAN.

In order to intuitively compare the FUIGN method with the Cycle-GAN method, the sample images generated by the FUIGN and Cycle-GAN methods are shown in Figure 6 and Figure 7. Figure 6 shows the sample images generated by the generator $G_{A2B}^{\left(F\right)}$ and $G_{A2B}^{\left(C\right)}$, respectively. Figure 7 shows the sample images generated by the generator $G_{B2A}^{\left(F\right)}$ and $G_{B2A}^{\left(C\right)}$, respectively. 

Figure 6 consists of two parts. The top of Figure 6 shows the generated results of the $G_{A2B}^{\left(F\right)}$ of the FUIGN method and the bottom of Figure 6 shows the generated results of $G_{A2B}^{\left(C\right)}$ of the Cycle-GAN method. As Figure 6 shows, the $G_{A2B}^{\left(F\right)}$ of FUIGN can generate the fuzzy image with the same blurring degree as the corresponding style image and retain complete structural information, which can meet the requirement of the training set. By comparison, the blurring degree of the outputs of the $G_{A2B}^{\left(C\right)}$ of Cycle-GAN is the same, which cannot meet the requirement of the training set. 

Figure 7 consists of two parts. The top of Figure 7 shows the generated results of the $G_{B2A}^{\left(F\right)}$ of the FUIGN method, and the bottom of Figure 7 shows the generated results of the $G_{B2A}^{\left(C\right)}$ of the Cycle-GAN method. As Figure 7 shows, the $G_{B2A}^{\left(F\right)}$ of FUIGN can generate the underwater clear image and retain complete structural information, which can meet the requirement. By comparison, the outputs of the $G_{B2A}^{\left(C\right)}$ of Cycle-GAN are still fuzzy, which cannot meet the requirement.

**Figure 8:**
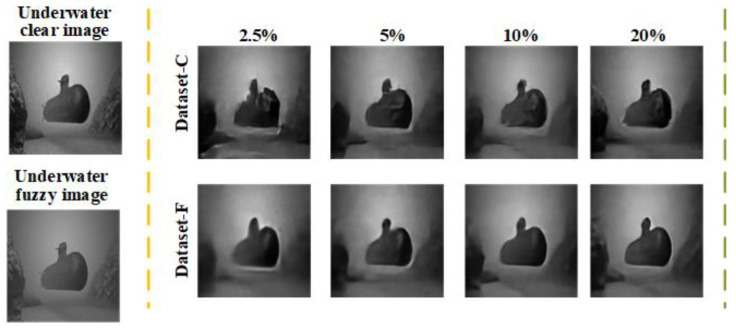
Comparison of simulation results of UGI-GAN trained with dataset-F and dataset-C at different sampling rates.

In order to verify the effectiveness of the paired underwater dataset (dataset-F) generated by FUIGN, a numerical simulation is performed. Furthermore, the UGI-GAN method [27] is trained with dataset-F and the paired underwater dataset (dataset-C) generated by Cycle-GAN, respectively, to compare the advantages and disadvantages of FUIGN and Cycle-GAN. The reconstructed results of UGI-GAN trained with dataset-F and dataset-C at varied sampling rates are shown in Figure 8. The clear submarine image is the target image for network training, and the fuzzy submarine image is used to simulate the M measurements collected by the bucket detector. Under the condition of the same sampling rate, the quality of the reconstructed results of the UGI-GAN method trained with dataset-F is better than the results trained with dataset-C. The simulation results show that dataset-F is more suitable than dataset-C to train UGI-GAN.

**Figure 9:**
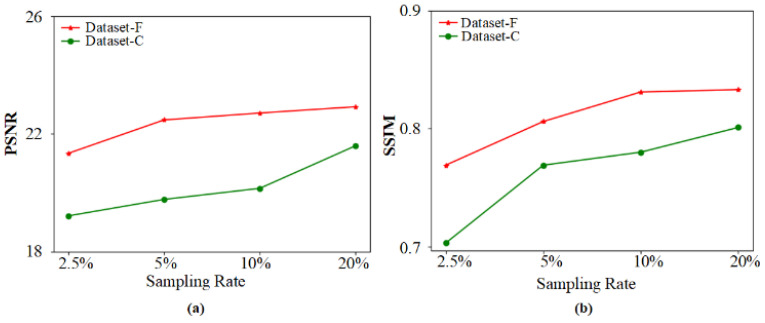
The PSNRs and SSIMs of the reconstructed submarine images of UGI-GAN trained with dataset-F and dataset-C at different sampling rates. (**a**) PSNRs of the reconstructed submarine images of UGI-GAN; (**b**) SSIMs of the reconstructed submarine images of UGI-GAN.

In order to compare the experimental results quantitatively, the SSIMs of PSNRs [41] of a reconstructed submarine of UGI-GAN trained with dataset-F and dataset-C at varied sampling rates are shown in Figure 9. The colored lines represent the reconstruction quality evaluation indexes of the reconstructed result of the corresponding dataset. The red and green lines represent dataset-F and dataset-C, respectively. The indexes of the reconstructed results of the UGI-GAN method trained with dataset-F are better than the results trained with the dataset-C under the condition of the same sampling rate. This indicates that the reconstructed quality of UGI-GAN trained with dataset-F is better than that of UGI-GAN trained with dataset-C. 

### 3.2. Numerical Simulations

**Figure 10:**
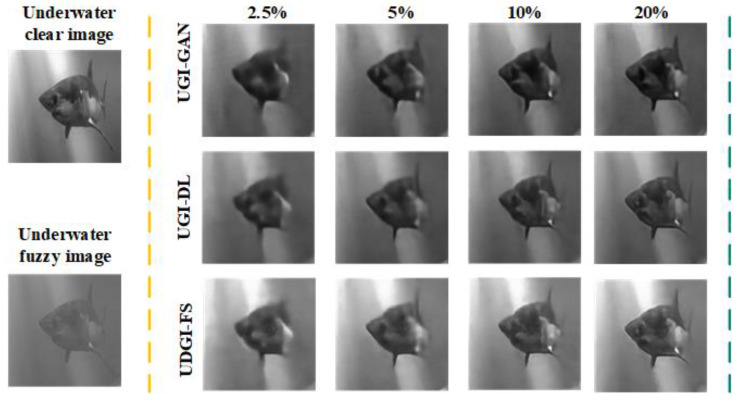
Comparison of reconstructed fish images of UDGI-FS, UGI-DL, and UGI-GAN at different sampling rates.

**Figure 11:**
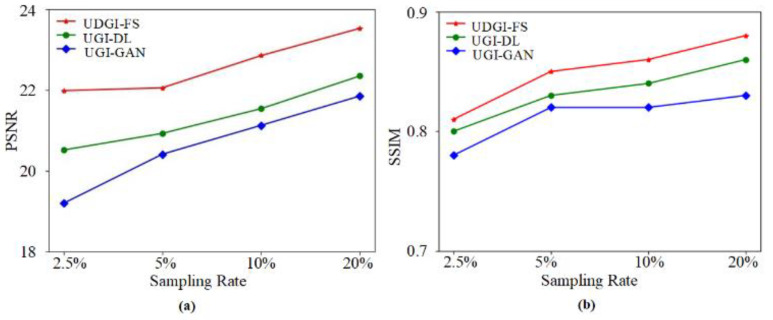
The PSNRs and SSIMs of the reconstructed fish images of UDGI-FS, UGI-DL, and UGI-GAN at different sampling rates. (**a**) PSNRs of the reconstructed fish images of UDGI-FS, UGI-DL, and UGI-GAN at different sampling rates; (**b**) SSIMs of the reconstructed fish images of UDGI-FS, UGI-DL, and UGI-GAN at different sampling rates.

In the simulation, the UGI-GAN [27] and UGI-DL methods trained with the underwater datasets generated by FUIGN are compared with the UDGI-FS method. The reconstructed images by the three methods at four different sampling rates, 20%, 10%, 5%, and 2.5%, are shown in Figure 10. The two images on the left of Figure 10 represent the paired underwater images. The clear fish image is treated as the target image for network training and the fuzzy fish image is used to simulate the measurements of the bucket detector. The reconstructed results of the three methods are shown on the right of the yellow dotted line in Figure 10. As Figure 10 shows, the quality of the reconstructed results of the three methods is getting better and better as the sampling rate increases from 2.5% to 20%. When the sampling rate is 2.5%, the UGI-GAN method cannot recover a clear fish image; it is still fuzzy, and the edge profile of the fish is distorted seriously. By comparison, the fish reconstructed by UGI-DL is clearer, which contains complete construction. In addition, the clarity degree of the fish reconstructed by UDGI-FS is better than that of UGI-DL. When the sampling rate increases from 5% to 20, the reconstructed results of UDGI-FS have high image contrast and a high clarity degree, which is better than those of the UGI-DL and UGI-GAN methods. In addition, the PSNR and SSIM curves of the reconstructed fish are shown in Figure 11. As Figure 11 shows, the UDGI-FS method performs best among the three methods. Figure 10 and Figure 11 show that the UDGI-FS network can reconstruct an image with high clarity degrees at a low sampling rate. 

**Figure 12:**
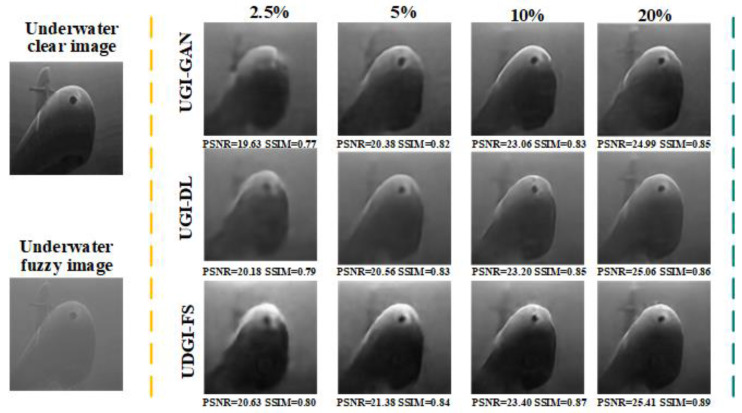
Comparison of reconstructed submarine images of UDGI-FS, UGI-DL, and UGI-GAN at different sampling rates.

To further verify the generalization ability of the network, a submarine image was selected for testing. The reconstructed results of UDGI-FS, UGI-DL, and UGI-GAN are shown in Figure 12. The PSNRs and SSIMs of reconstructed images were calculated. As Figure 12 shows, the reconstructed submarine of UDGI-FS has a relatively complete profile and high clarity degree at the sampling rate of 2.5%, which is better than UGI-DL and UGI-GAN. With the increase in the sample rate, the quality of the reconstructed submarine of UDGI-FS becomes better and better, which is better than the other two methods. The generalization test results verify that the proposed UDGI-FS method has the best generalization ability among the three methods. 

## 4. Experimental Methods

### 4.1. Experimental Setup

**Figure 13:**
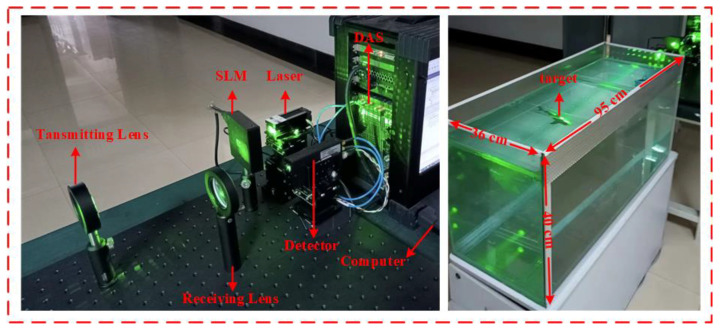
Experimental setup implemented in the lab.

Based on Figure 1, the schematic of the UDGI-FS system, an experiment setup was implemented. The experiment setup in the lab is shown in Figure 13. As Figure 13 shows, the laser source (OEM-I-532), the wavelength of which is in the range of 510 nm and 530 nm, is a pulsed laser and its average pulse power is 5mW, which indicates it is a viable choice of light source. Firstly, the emitted laser, of which the spot diameter is 10 mm, is directed into the SLM (LC 2012) controlled by the computer. The spatial distribution of the laser is modulated by the SLM. Then, the modulated laser light passes through the transmitting lens, the diameter of which is 50 mm, to illuminate the underwater target in the sink. Then, the reflected light passes the receiving lens, the diameter of which is 50 mm. The reflected light is collected by the bucket detector, the model number of which is H11706P-01. The data acquiring system (DAS), the model number of which is M4x.4450-x4, is used to record the total light intensity. Finally, the total light intensity is transferred to the computer for imaging. In the experiment, the toy shark and submarine are placed in a sink, the size of which is 95 cm × 36 cm × 40 cm. In order to reduce the effect of environment on imaging quality, the experiment was performed in a dark environment to avoid interference from background. 

### 4.2. Experimental Results

**Figure 14:**
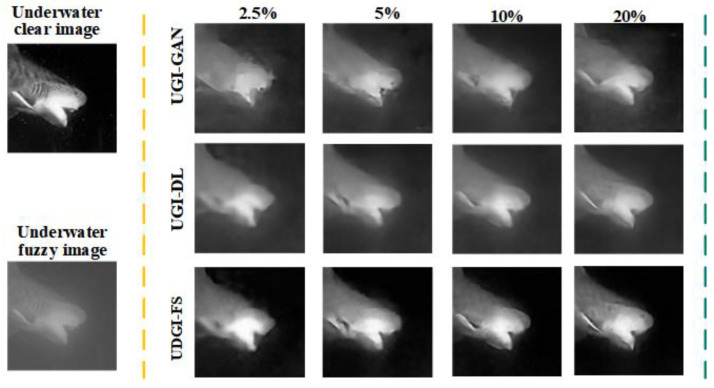
Comparison of reconstructed shark images of UDGI-FS, UGI-DL, and UGI-GAN at different sampling rates.

**Figure 15:**
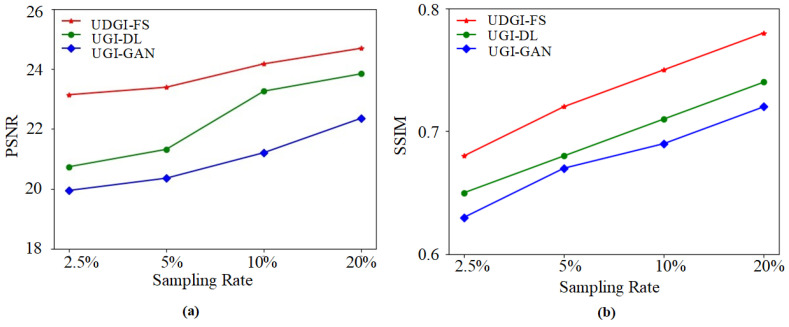
The PSNRs and SSIMs of the reconstructed shark images of UDGI-FS, UGI-DL, and UGI-GAN at different sampling rates. (**a**) PSNRs of the reconstructed shark images of UDGI-FS, UGI-DL, and UGI-GAN at different sampling rates; (**b**) SSIMs of the reconstructed shark images of UDGI-FS, UGI-DL, and UGI-GAN at different sampling rates.

The trained UDGI-FS model was used to recover the underwater shark images from the measurements of the bucket detector under different sampling rates. The experimental results are shown in Figure 14. As Figure 14 shows, the reconstructed shark of UDGI-FS is relatively clear at the sampling rate of 2.5%, which is better than the UGI-DL and UGI-GAN methods. With the increasing of the sampling rate, the quality of the results of the UDGI-FS method is the best of the three methods. In addition, the PSNR and SSIM curves of the reconstructed shark are shown in Figure 15. As Figure 15 shows, UDGI-FS performs the best among the three methods. Figure 14 and Figure 15 show that the proposed UDGI-FS method has a good ability of deblurring at a low sampling rate, which is better than the UGI-DL and UGI-GAN methods. 

**Figure 16:**
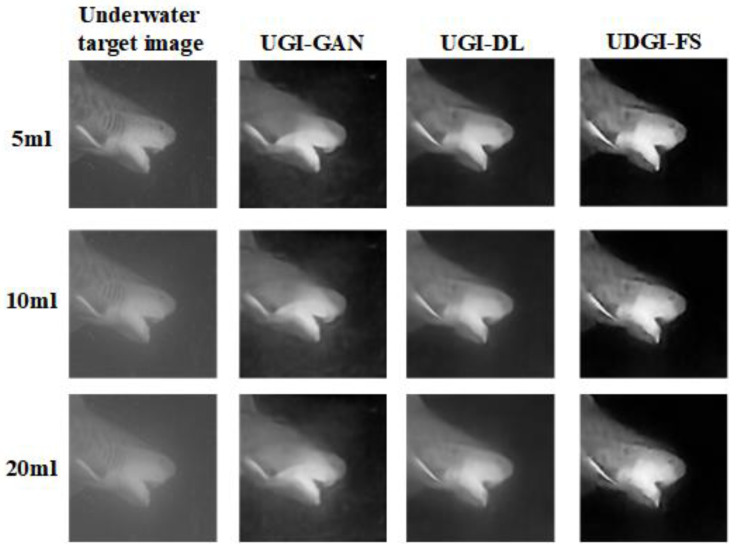
Comparison of reconstructed shark images of UDGI-FS, UGI-DL, and UGI-GAN at different degrees of underwater blurring.

**Figure 17:**
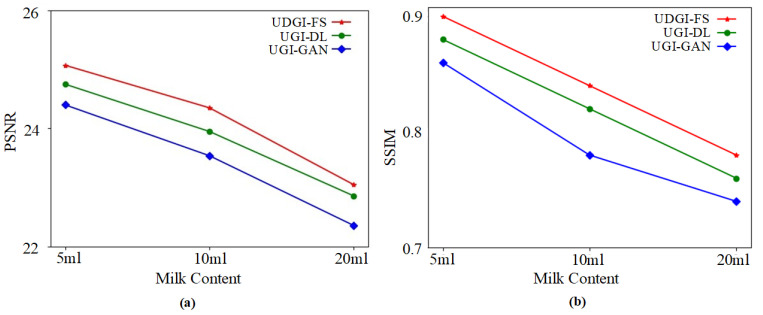
The PSNRs and SSIMs of the reconstructed shark images of UDGI-FS, UGI-DL, and UGI-GAN at different degrees of underwater blurring. (**a**) PSNRs of the reconstructed shark images of UDGI-FS, UGI-DL, and UGI-GAN at different degrees of underwater blurring; (**b**) SSIMs of the reconstructed shark images of UDGI-FS, UGI-DL, and UGI-GAN at different degrees of underwater blurring.

A shark model was used to examine the reconstruction ability of the UDGI-FS model for the underwater environment with different blurring degrees. As Figure 16 shows, the three methods are trained at a 20% sampling rate. In order to obtain different blurring degrees, 5 mL, 10 mL, and 20 mL of milk were poured into the sink, respectively. As Figure 16 shows, the blurring degree has a great influence on the reconstructed results. As Figure 16 shows, the reconstructed quality of the proposed method becomes better and better with the decreasing of the blurring degree. In the underwater environment mixed with 20 mL milk, UDGI-FS can still reconstruct a clear shark image, which is better than the UGI-DL and UGI-GAN methods. As the quantity of milk poured into the sink decreases, the quality of the reconstructed results of UDGI-FS becomes better and clearer, which is better than the UGI-DL and UGI-GAN methods. In addition, the PSNR and SSIM curves of the reconstructed shark at different underwater blurring degrees are shown in Figure 17. As Figure 17 shows, UDGI-FS performs the best among the three methods. Figure 16 and Figure 17 show that that the UDGI-FS method can achieve high-quality image reconstruction at a high blurring degree, and the reconstruction ability is better than the UGI-DL and UGI-GAN methods. 

**Figure 18:**
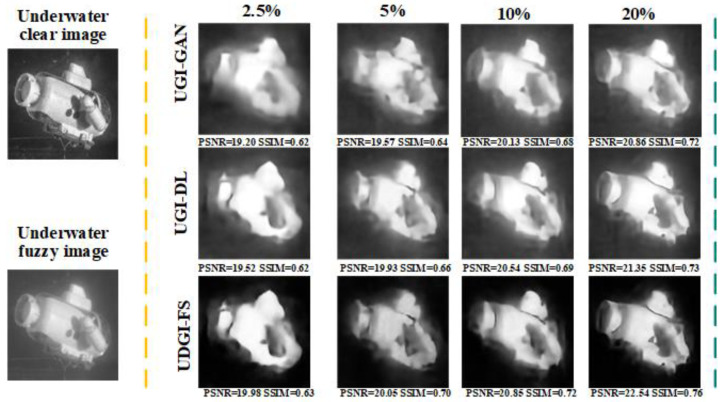
Comparison of reconstructed submarine images of UDGI-FS, UGI-DL, and UGI-GAN at different sampling rates.

To further verify the generalization ability of the network, an underwater submarine model was selected for the experiment. The reconstructed results of the UDGI-FS, UGI-DL, and UGI-GAN are shown in Figure 18. The PSNRs and SSIMs of the reconstructed images were calculated. It can be seen that UDGI-FS performs the best among the three methods. It can be concluded that the proposed UDGI-FS has good generalization ability under a low sampling rate and the submarine can be reconstructed in high quality.

**Figure 19:**
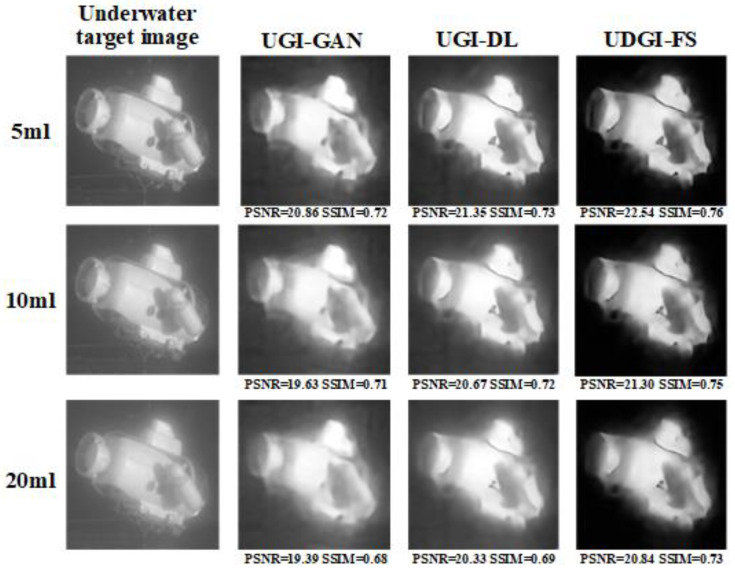
Comparison of reconstructed submarine images of UDGI-FS, UGI-DL, and UGI-GAN at different degrees of underwater blurring.

An underwater submarine model was used to examine the reconstruction ability of the UDGI-FS model for the underwater environment with different blurring degrees. As Figure 19 shows, the three methods were trained at a 20% sampling rate. The PSNRs and SSIMs of the reconstructed images were calculated. It can be seen that UDGI-FS can achieve high-quality image reconstruction with a high blurring degree, and the reconstruction ability is better than the UGI-DL and UGI-GAN methods.

## 5. Conclusions

In conclusion, the FUIGN method based on the few-shot learning method is proposed in order to obtain paired underwater datasets meeting the requirements for training the reconstruction network. As the underwater fuzzy image is hard to obtain, an asymmetric dataset containing 15,000 underwater clear images and 1500 underwater fuzzy images was used in the training process. The simulation results show that the paired underwater datasets generated by the generator $G_{A2B}^{\left(F\right)}$ of FUIGN are better than that of the Cycle-GAN method. In addition, to reconstruct high-quality results, UDGI-FS is proposed. The UDGI-FS consists of two parts: UGI-DL and $G_{B2A}^{\left(F\right)}$. UGI-DL is used to reconstruct the underwater image, and $G_{B2A}^{\left(F\right)}$ is used to decrease the blurring degree of the reconstructed results of UGI-DL. In the UGI-DL model, the generator utilizes modified U-Net with the res-net block as the main network architecture and adds double skip connections between corresponding layers. In the double skip connections, the attention gate is added to each of the skip connections to improve the reconstruction ability of the network. 

The performance of dataset-F generated by FUIGN is analyzed and compared with dataset-C generated by Cycle-GAN through numerical simulations. Simulation results indicate that dataset-F has better performance than dataset-C. To compare the qualities of the reconstructed results of UGI-GAN trained with the two datasets, the PSNRs and SSIMs were also calculated. The quality indexes demonstrate that dataset-F has better performance in training the reconstruction network. In addition, the performance of the proposed UDGI-FS method is analyzed through numerical simulations and experiments. Experimental results indicate that the UDGI-FS method has better reconstruction performance than the UGI-GAN and UGI-DL methods. In addition, the PSNRs and SSIMs of the reconstructed results demonstrate that the UDGI-FS method has the best reconstruction ability and generation ability. Meanwhile, experimental results show that the reconstructed image of UDGI-FS has the best visual effect in underwater environments with different blurring degrees. Experimental results show that the FUIGN method can provide a promising method for generating paired underwater datasets. In addition, UDGI-FS can be used to reconstruct the high-quality underwater results. 

## Figures and Tables

**Table 1:** The training and reconstruction durations of UDGI-FS, UGI-DL, and UGI-GAN at 20% sampling rate.

Method	Training Duration	Reconstruction Duration
UDGI-FS	24 h	65 ms
UGI-DL	24 h	35 ms
UGI-GAN	23 h	25 ms

## Data Availability

The data that support the findings of this study are openly available in UDGI-FS at https://github.com/yu-zhongyang/UDGI.git [accessed on 5 August 2022].
